# Gut Microbiota Targeted Approach in the Management of Chronic Liver Diseases

**DOI:** 10.3389/fcimb.2022.774335

**Published:** 2022-04-04

**Authors:** Jing Liu, Dakai Yang, Xiaojing Wang, Paul Tetteh Asare, Qingwen Zhang, Lixin Na, Lei Shao

**Affiliations:** ^1^ Department of Research, Shanghai University of Medicine and Health Sciences Affiliated Zhoupu Hospital; The College of Medical Technology, Shanghai University of Medicine and Health Sciences, Shanghai, China; ^2^ Key Laboratory of Medical Science and Laboratory Medicine, School of Medicine, Jiangsu University, Zhenjiang, China; ^3^ School of Pharmacy, Shanghai University of Medicine and Health Sciences, Shanghai, China; ^4^ Human and Animal Health Unit, Department of Epidemiology and Public Health, Swiss Tropical and Public Health Institute, Basel, Switzerland

**Keywords:** gut microbiota, chronic liver diseases, bile acids, choline, probiotics, prebiotics

## Abstract

The liver is directly connected to the intestines through the portal vein, which enables the gut microbiota and gut-derived products to influence liver health. There is accumulating evidence of decreased gut flora diversity and alcohol sensitivity in patients with various chronic liver diseases, including non-alcoholic/alcoholic liver disease, chronic hepatitis virus infection, primary sclerosing cholangitis and liver cirrhosis. Increased intestinal mucosal permeability and decline in barrier function were also found in these patients. Followed by bacteria translocation and endotoxin uptake, these will lead to systemic inflammation. Specific microbiota and microbiota-derived metabolites are altered in various chronic liver diseases studies, but the complex interaction between the gut microbiota and liver is missing. This review article discussed the bidirectional relationship between the gut and the liver, and explained the mechanisms of how the gut microbiota ecosystem alteration affects the pathogenesis of chronic liver diseases. We presented gut-microbiota targeted interventions that could be the new promising method to manage chronic liver diseases.

## Introduction

Liver disease accounts for approximately 2 million deaths per year worldwide, liver disease is currently the 11^th^ leading cause of mortality worldwide (Asrani et al., 2019), and it caused 4.6% of deaths in the Asia-Pacific region in 2015, compared with 2.7% of the USA (Younossi et al., 2020) and 2.1% in Europe (Sarin et al., 2020). Chronic liver disease includes chronic viral infection, alcoholic and non-alcoholic fatty liver diseases (ALD/NAFLD), non-alcoholic steatohepatitis (NASH), primary biliary cholangitis, cirrhosis, and hepatic encephalopathy (HE). Metabolic toxic, autoimmune, viral, and genetic disorders are clinical risk factors for chronic liver diseases (Rinella, 2015). Nutrition is also an vital factor for chronic liver disease (Johnson et al., 2013). For example, many patients who suffer from advanced liver disease have malnutrition problems as well. Malnutrition causes changes in the gut microbiota, leading to dysbiosis, and bacterial and/or pathogen-derived factors may be transferred from the gut to the liver. The gut microbiota contributes to the progression of chronic liver disease as well as hepatocellular carcinoma. The intestinal permeability changes are associated with gut microbiota (GM) involved in developing chronic liver disease (Milosevic et al., 2019). The gut microbiota closely interacts with a variety of immune cells in the liver (macrophages, Natural killer T cells, γδ T cells) (Yang et al., 2021). The pro-inflammatory factors generated from gut microbiota such as peptidoglycan and lipopolysaccharide (LTA) (Lebeer et al., 2010) induce excessive activation of immune cells and thus may aggravate the liver injury, inflammation, which worsen the chronic liver disease (Wheeler, 2003). On the contrary, metabolites from gut bacteria such as tryptophan metabolites, acetic acid, butyric acid, bile acids, phenolic compounds, and carotenoids may restore oxidative damage, inflammatory responses and lipogenesis in the liver tissue (Molinero et al., 2019). Many dietary components have the hepatoprotective function, the components could interact with gut microbiota and gut-derived metabolites, and improve intestinal mucosal immunity (Li et al., 2019), increase the beneficial bacteria composition, and reduce toxic metabolites release (Chen et al., 2014). Some natural products such as flaxseed oil (Zhang et al., 2017), brown algae *Lessonia nigrescens* (Zhao et al., 2017), and the herbal medicine *Qushi Huayu* decoction can revert gut dysbiosis and improve liver disease symptoms (Feng et al., 2017).

There is a close connection between the intestine and the liver through the gut microbiota and their metabolites, but the mechanism of how gut microbiota affect chronic liver diseases is still not clear. In this review article, we discussed in detail how the intestine and the liver communicate with each other through bile acid circulation and choline metabolites, elaborated the mechanism of the gut microbiota change that affects chronic liver disease. All relevant articles until May 2021 were included. The articles were searched through PubMed. We searched using terms including “gut microbiota”, “gut dysbiosis”, “liver” and “steatosis” as well as “non-alcoholic fatty liver disease”, “steatohepatitis”, “diets”, “probiotics” and “primary sclerosing cholangitis”. Over 100 articles were included, based on great deal of research works, we give a new promising approach that alters the structure of gut microbiota that could be used to manage the chronic liver disease.

## Bi-Direction Communication Between the Gut and the Liver

### The Association Between the Disorder of Intestinal Barrier Function and Liver Disease

Since the intestine and the liver have a close connection in anatomy and physiology, intestinal permeability and intestinal microbiota are closely connected to chronic liver disease (Chopyk and Grakoui, 2020). The rise in the incidence of liver diseases is a positive association with gastrointestinal and immune disorders (Brenner et al., 2015; Fukui, 2019). The intestinal barrier consists of physical, immune, and microbial components. The physical barrier is related to epithelium and mucus elements (Spadoni et al., 2017). A thick layer of mucus covered the whole intestinal epithelium, which contains mucin (MUCs), a highly glycosylated glycoprotein produced by goblet cells (Cornick et al., 2015). MUCs contain two types: the secreted mucins (MUC2) and membrane-bound mucins (MUC1, MUC3, MUC4) (Johansson et al., 2011). Mucus can act as a physical barrier to prevent pathogens invasion, they also can be used as a carbohydrates source for symbiotic bacteria. The most important function of mucus is immunity activity. Mucus contains many immunomodulatory molecules, such as anti-microbial peptides and immunoglobulins (Birchenough and Johansson, 2020). The thickness of the mucus layer also affects the survival and proliferation of bacteria (Schroeder, 2019). Bacteria also can affect mucus development in the intestine. In the first few weeks of germ-free mice experiencing bacterial colonization, the levels of IgA in the mucus were found to have a peak. The composition of bacteria in the intestine also has a significant alteration: the number of the *Bacteroidetes* increased while the *Firmicutes* reduced (Johansson et al., 2015). After five weeks of bacteria colonization, the mucus quality and microbiota composition of germ-free mice returned similar to that of wild-type mice, which indicates mucus could maintain the small intestine homeostasis.

**Figure 1:**
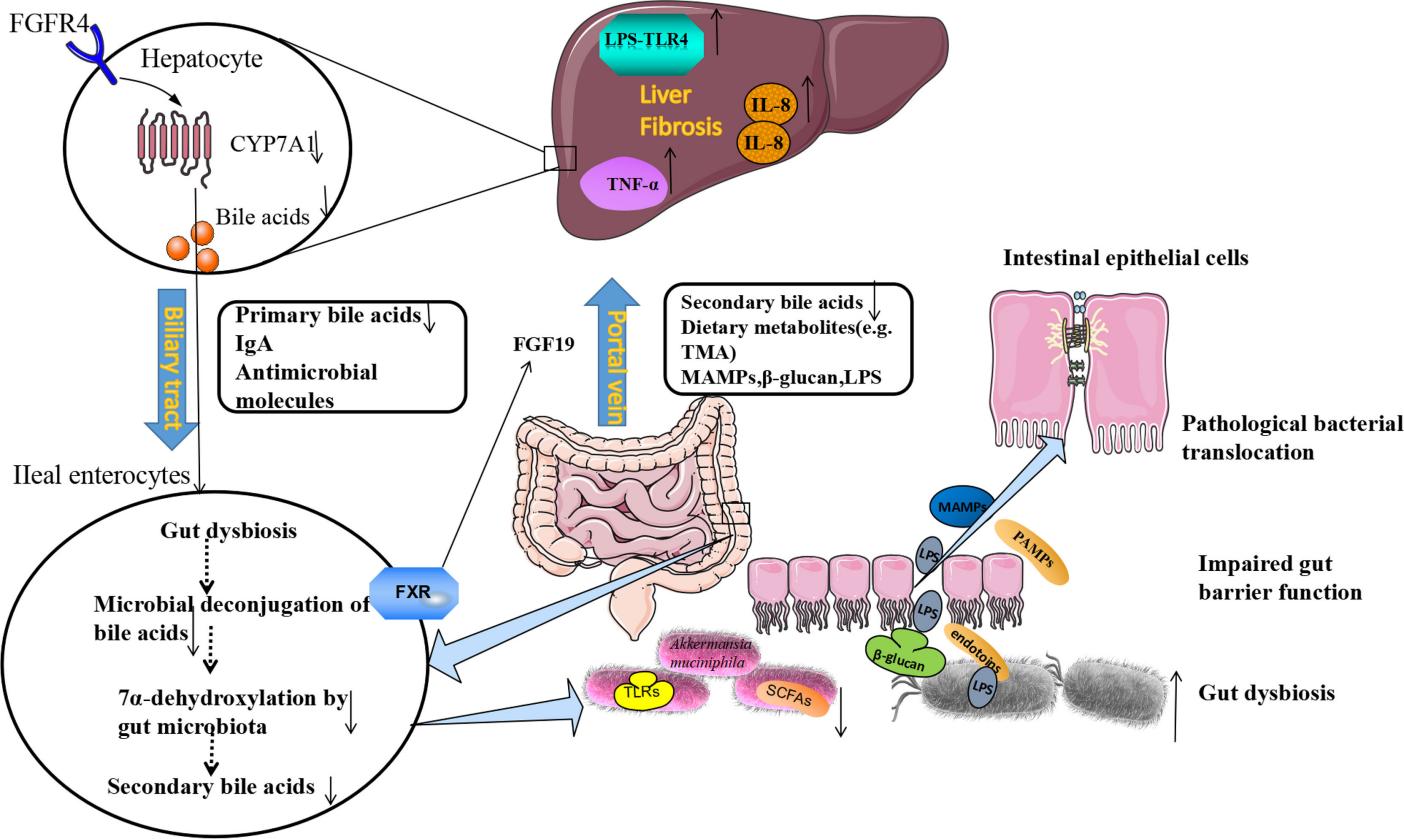
The Etiological Mechanism of bi-direction circulation between the intestine and the liver. The liver communicates with the gut through the portal veins, bile duct, and circulation system. Under pathological conditions of the liver, the gut microbiota dysbiosis could be found. The beneficial bacteria such as *Akkermansia muciniphila* decreased, and pathogenic bacteria such as *Enterobacteriaceae* increased. Following the production of SCFAs (butyrate) decreased, the production of antimicrobial molecules (IgA) reduced, tight junction integrity was disrupted, and intestinal permeability increased. LPS, β-glucan, and MAMPs moved from the gut lumen into the portal circulation, and translocate to the liver through portal veins. LPS activates pathogen recognition receptor TLR4 on liver resident macrophages Kupffer cells and hepatic stellate cells. The TLR4 signal in Kupffer cells triggers a downstream inflammatory cascade reaction to mediate the activation of TNF-α and IL-8. The TLR4 signal also promotes fibrosis in hepatocytes. On the other hand, fibrosis alters bile acid homeostasis and contributes to gut dysbiosis. Gut bacteria can express bile salt hydrolases to deconjugate primary bile acids. The 7α-dehydroxylase expressed by other bacteria can convert primary bile acids to secondary bile acids. In fibrosis, these bacteria decreased and reduced the conversion. Bile acids produced from the gut could bind to FXR, and produce FGF19, which reach the liver through the portal vein and down-regulates the synthesis of primary bile acids in hepatocytes by inhibiting cholesterol 7α-monooxygenase (CYP7A1) expression, further contributing to gut dysbiosis. lgA, immunoglobulin A; FXR, farnesoid X receptor; MAMPs, microbial-associated molecular patterns; SCFAs, short-chain free acids; LPS, lipopolysaccharide; TLR4, toll-like receptor 4; FGF19, fibroblast growth factor 19.

Furthermore, pathogen recognition receptor toll-like receptors (TLRs) are important mediators between microorganisms and host. They play important role in mucus formation (Rakoff-Nahoum et al., 2004). Commensal microbes can provide continuous stimulation of TLRs on enterocytes to increase MUCs and anti-microbial peptides production, which maintain the tight junction of the epithelial barrier (Mukherjee and Hooper, 2015). Intestinal dysbiosis could increase intestinal permeability and disrupt the tight junction between intestinal epithelial cells. Microbial-derived metabolites, such as β-glucan, endotoxins (lipopolysaccharide, LPS) and bacterial viral RNAs, these molecules we called pathogen-associated molecular patterns (PAMPs). PAMPs can disturb the gut-liver axis, cause gut dysfunction and intestinal dysbiosis, which will cause intestinal permeability to increase in liver cirrhosis and small intestinal bacterial overgrowth (Anand et al., 2016; Fukui, 2021). Through the portal vein, blood flows from the intestine to the liver, PAMPs activate TLR4 on liver macrophages and hepatic immunity cells (Uesugi et al., 2001). The TLR4 signal in liver resident macrophages triggers a downstream inflammatory reaction to mediate the activation of TNF-α and IL-8 (Seki and Schnabl, 2012). The TLR4 signal also promotes fibrosis in hepatosplenic cells by downregulating the membrane-binding inhibitor homologous BAMBI (Lebeaupin et al., 2015), ultimately causing hepatic injury (Anand et al., 2016) (**Figure 1**).

### Intestine and Liver Communicates Through Bile Acids Enterohepatic Circulation

The liver communicates with the gut through the portal veins, bile duct, and circulation system (Stärkel and Schnabl, 2016). In the gut, the microbiota can metabolize the substance from diet and host metabolism, the gut microbiota metabolites can be translocated to the liver and affect liver functions (Tripathi et al., 2018). The liver can also release bioactive mediators to affect the gut system through the biliary tract and the circulation system, the most common mediators are bile acids (BAs) (Chiang, 2013). Bile acids are synthesized by the pericentral hepatocytes through the conversion of cholesterol, and primary BAs are conjugated with taurine and glycine in a ratio of 1:3 in humans which are released in the biliary tract (Wahlström et al., 2016). The enterohepatic circulation of BAs plays an important physiological role in the absorption of dietary nutrients, lipids, toxic metabolites, and exogenous substances excreted with bile digestion (Wahlström et al., 2016). After entering the intestinal tract, about 95% of the BAs are actively reabsorbed into epithelial cells of the terminal ileum and transported back to the liver through ileal bile acid transporter protein (IBAT). About 5% of BAs are metabolized by the gut microbiota to secondary bile acids, which increases the diversity and hydrophobicity of bile acids, and is beneficial for the excretion of bile acids (Arab et al., 2017). The lipid digestion needs a large number of bile acids, the liver can recycle and recover the BAs through enterohepatic circulation, which can reuse limited bile acids as well as exchange metabolites between the intestine and the liver (Arab et al., 2017).

By activating specific receptors expressed in different cell types can trigger the physiological action of BAs, and then the metabolism of organisms is regulated. FXR is the most important receptor involved in regulating the intestinal-hepatic circulation of BAs and BAs biosynthesis, and it is expressed in the liver, intestine, kidney, and other tissues (Zarrinpar and Loomba, 2012). FXR receptor is also an inhibitor of BAs synthesis in the liver, which regulates BAs metabolism in enterohepatic circulation and can effectively control BAs content in the liver and intestine. In the intestines, BAs stimulates the FXR receptor, activates the FXR-FGF15/19 signaling pathway, mediates the expression of fibroblast growth factors (Fibroblast Growth Factor, FGF15/19) in the epithelial cells of the intestine, and thus affects the secretion of the FGFR4 receptor (Fibroblast Growth Factor Receptors 4) (Copple and Li, 2016). FGF15/19 reaches the liver and down-regulates the synthesis of BAs in hepatocytes by inhibiting cholesterol 7α-monooxygenase (CYP7A1) (Sinal et al., 2000), forming a feedback system that regulates BAs production, as shown in **Figure 1** (Broeders et al., 2015). This provides feedback that if sufficient bile acids are absorbed in the ileum, new bile acids synthesis in the liver is inhibited.

BAs regulates the ecosystem of gut microbiota and the interaction between the BAs and GM is a two-way direction (Ridlon et al., 2016). Studies have shown that bile ducts blockages prevent bile from flowing into the intestines, which will cause the small intestinal bacterial overgrowth and translocation, and this could be reversed by BAs administration. BAs binding to FXR could induce antimicrobial peptides (AMPS) production, which has a function in inhibiting the intestinal microbial overgrowth and intestinal barrier dysfunction (Jiang et al., 2015). Intestinal gut microbiota dysbiosis disrupts the balance between primary and secondary BAs (Ridlon et al., 2014). The imbalance between BAs and intestinal bacteria leads to a series of host immune responses related to liver disease progression, bile acids regulate specific host metabolic pathways and modulate the inflammatory responses through FXR and G protein-coupled bile acid receptor 1 (Marialena et al., 2016). Gut microbes can also use bile acids to regulate the aggregation of NKT cells in mouse livers (Ma et al., 2018).

### Choline Metabolites Affect Liver Diseases

Choline, especially phosphatidylcholine, plays an important role in transporting lipids of the liver. In the absence of choline, fat accumulates in the liver, which results in NAFLD. It is worth noting that rodents fed with a choline-deficient diet are often being used as models of non-alcoholic steatohepatitis (NASH) (Valor et al., 2019). Choline is not only important for liver function but also protects brain memory, enhances the brain to concentrate, improves motor ability, and delays the aging of the brain (Derbyshire and Obeid, 2020). Choline can be converted to phosphatidylcholine (lecithin) by the host to assist the liver in excreting very-low-density lipoprotein (VLDL) particles (Mehedint and Zeisel, 2013). This process prevents triglycerides accumulation in the liver. However, gut microbiota such as *Firmicutes* and *Proteobacteria* can convert choline into trimethylamine (TMA); TMA can be transferred and converted to harmful metabolite trimethylamine N-oxide (TMAO) in the liver (Coutinho-Wolino et al., 2021). The importance of methylamine is increasingly recognized, involving the liver, heart metabolism, and neurological disorders (Chhibber-Goel et al., 2016). Increased systemic circulation of TMAO is accompanied by a decreased level of phosphatidylcholine produced by the host (Del Rio et al., 2017). This imbalance is characteristic of intestinal disorders of patients. In human and experimental NAFLD models, TMAO is associated with liver injury due to increased triglyceride accumulation/hepatic steatosis (Gogiashvili et al., 2016; Sherriff et al., 2016; Del Rio et al., 2017).

### Free Fatty Acids and Their Roles on Liver Disease

Free fatty acids include short-chain fatty acids (SCFAs) and long-chain fatty acids (LCFAs). SCFAs mainly include acetate, propionate, and butyrate, which are produced during the bacterial fermentation of dietary fibers (Bergman, 1990). Butyrate could be used as the energy source of intestinal cells and helps to maintain the intestinal barrier functions (Hamer et al., 2010). Alcohol-induced liver injury is characterized by butyrate and propionate decreased, the level of acetate increased (Peng et al., 2014). Butyrate enhances the immune barrier of the intestinal mucosa, which prevents bacteria and their metabolites from entering the bloodstream and reducing the inflammatory response. Butyrate supplementation in glyceryl tributyl ester can reduce intestinal permeability disrupted by alcohol (Cresci et al., 2017). However, the mechanism through which tributyl glycerol protects the intestinal barrier remains unknown. LCFAs were also found closely connected with alcohol-induced liver injury. In the mice fed with an alcohol diet, the production of C15:0 and C17:0 was significantly decreased compared to the control fed with an isocaloric diet (Shi et al., 2015). In general, the level of total saturated LCFAs is positively correlated with the lumen abundance of *Lactobacilli* (known metabolizers of saturated LCFAs). Saturated LCFA can promote colonic motility in rats and increase stool frequency (Zhao et al., 2018). *Lactobacillus rhamnosus* administration can increase the levels of luminal LCFA, and enhance its probiotic effects (Shi et al., 2015).

## Specific Gut Microbiota Altered in Different Types of Chronic Liver Diseases

Alterations of gut microbiota compositions in different types of chronic liver diseases were summarized in **Table 1**.

**Table 1:** Alterations of gut microbiota compositions associated with Chronic Liver Disease.

Comparison^a^	Microbiota	Sample	Mechanism	Ref.
NASH patients vs Healthy control	*Bacteroides* ↑*Proteobacteria*↑ *Enterobacteriaceae*↑*and Escherichia*↑*Firmicutes*↓ *Actinobacteria*↓*Klebsiella Pneumoniae*↑	stool	alcohol‐producing bacteria increased, supply a constant source of ROS, liver inflammation increased	(Duarte et al., 2019; Yuan et al., 2019)
ALD patients vs Healthy control	*Proteobacteria*↑*Bacteroidetes*↓*Firmicutes*↓ *Enterobacteriaceae*↑ *Bacteroidetes*↓and *Lactobacillus*↓	colon contents	Beneficial bacteria decreased, intestinal permeability increased, bacterial endotoxins exposure systemic	(Mutlu et al., 2012)
Severe AH patients vs Healthy control	*Bifidobacteria*↑ *Streptococci* ↑*Enterobacteria*↑ *Clostridium leptum* ↓ *Faecalibacterium prausnitziithan* ↓	stool	anti-inflammatory bacteria decrease, intestinal dysbiosis caused gut permeability, facilitates microbiota translocation	(Llopis et al., 2016)
Severe AH mice vs non-AH mice	*Bilophila, Alistipes*↑*, Butyricimonas* ↑, *Clostridium cluster XIVa*↑ *Parasutterella excrementihominis*↓	stool	pro-inflammatory cytokine producing bacteria increased, anti-inflammatory bacteria decreased	(Llopis et al., 2016; Jefferies et al., 2018)
HBV patients vs Healthy control	*Fusobacteria*↑*Veillonella*↑*Haemophilus*↑ *Prevotella* ↓ *Phascolarctobacterium*↓	stool	pathogens colonization in the gut, promote systemic inflammation and worsen hepatic dysfunction	(Chen et al., 2020)
HCV patients vs Healthy control	Phylum:*Bacteroidetes*,Firmicutes↓ Genera:*Prevotella*↑*Acinetobacter*↑ *Phascolarctobacterium*,↑ *Veillonella*↑*Faecalibacterium*↑ *Ruminococcus*↓, *Clostridium*↓ and *Bifidobacterium*↓	stool	HCV infection induced intestine dysbiosis; carbohydrates concentrations increased by impairment in the intestine and the expansion of bacteria	(Aly et al., 2016)
PSC patients vs Healthy control	*Enterococcus*↑, *Fusobacterium*↑*and Lactobacillus*↑	stool	serum alkaline phosphatase increased	(Sabino et al., 2016)
PSC patients vs Healthy control	*Clostridiales* II ↓*Veilonella* ↑	stool	*Veilonella* have pro-inflammatory features	(Van den Bogert et al., 2014; Rossen et al., 2015; Iwasawa et al., 2017)
PSC patients vs Healthy control	*Blautia* ↑ *Barnesiellaceae* ↑*Proteobacteria* ↑*Parabacteroides*↑	Colon contents	*Blautia* can convert primary to secondary bile acids in the intestine., *Proteobacteria* and *Parabacteroides* are bile-tolerant taxons	(Torres et al., 2016; Rühlemann et al., 2019)
PSC patients vs Healthy control	*Escherichia* ↑*Lachnospiraceae* ↑ *Megasphera* ↑ *Prevotella* ↓ *Roseburia* ↓*Bacteroide*s ↓	stool	*Bacteroidetes* is associated with a reduced level of secondary bile acids and play an important role in bile acid deconjugation	(Quraishi et al., 2017)
Cirrhosis patients vs Healthy control	*Bifidobacterium*↓ *Bacteroidetes* ↓ *Proteobacteria* ↑ *Fusobacteria*↑ *Enterobacteriaceae*↑ *and Enterococcus*↑	stool	*Enterobacteriaceae* release endotoxin, damage the barrier function of the intestine, and increase the intestinal permeability	(Chen et al., 2011; Liu et al., 2012)
Cirrhosis patients vs Healthy control	*Enterobacteriaceae*↑*Alcaligenaceae*↑*Streptococcaceae*↑ *Veillonellaceae*↑and *Fusobacteriaceae*↑*Bacteroidetes*↓ *Ruminococcaceae*↓ *and Lachnospiraceae* ↓	stool	pathogenic taxa overgrowth is associated with disease progression and endotoxemia, the reduction taxa can produce SCFAs and anti-bacterial peptides.	(Bajaj et al., 2014b; Trebicka et al., 2021)
HE patients vs Healthy control	*Megasphaera*↑*Enterococcus*↑ *and Burkholderia*↑*Veillonellaceae* ↑ *Fecalibacterium* ↓*Blautia* ↓*, Roseburia* ↓*and Dorea* ↓	stool	Decreased bacteria associated with good cognition and decreased inflammation, increased bacteria linked to poor cognition and inflammation	(Bajaj, 2014; Bajaj et al., 2016; Bajaj et al., 2019)
HCC patients vs cirrhosis	*Escherichia coli* ↑	stool	hepatocarcinogenesis attributed to the *E. coli* overgrowth	(Grt et al., 2016)
HCC rats vs Healthy control	*Lactobacillus* ↓*Bifidobacterium* ↓*and Enterococcus* ↓	stool	Intestinal inflammation increased, probiotics decreased, which can inhibit the translocation of endotoxin, activation of DAMPs, reduce tumorigenic inflammation	(Zhang et al., 2012)

a A comparison of condition A vs condition B; ↑, increase in condition A related to condition B; ↓, decrease in condition A related to condition B.

### Non-Alcoholic Fatty Liver Disease

NAFLD is characterized by fat deposits in the liver and lipid metabolism disorders without significant alcohol consumption (Angulo, 2002). NAFLD can develop into non-alcoholic steatohepatitis (NASH), fibrosis, cirrhosis, and even hepatocellular carcinoma (HCC) (Sun et al., 2015). NAFLD has been considered as a multidirectional relationship with metabolic syndrome (Godoy-Matos et al., 2020). The prevalence of NAFLD ranges from 25% to 45%, and NAFLD is the most common cause of liver damage worldwide (Bellentani et al., 2010). According to GHE data in 2015, NAFLD accounted for 11.7% of all deaths from liver cirrhosis and other chronic liver diseases (Kuruvilla et al., 2016). Another study found that the prevalence of NAFLD was 46%, and biopsy confirmed the prevalence of NASH was as high as 12% (Williams et al., 2011). Lifestyles and dietary habits are closely related to the prevalence of NAFLD. There is increasing evidence that NAFLD patients also have gut microbiota dysbiosis problems. A few microbiotas could be used as biomarkers to distinguish healthy individuals from patients with NAFLD, NASH, or cirrhosis (Aron-Wisnewsky et al., 2020). Interestingly, it is the degree of cirrhosis, but not the NASH presence, which is related to intestinal leakage and intestinal bacterial overgrowth (Miele et al., 2009). The abundance of *Bacteroides* increased significantly and *Firmicutes* decreased in NASH groups when compared with the healthy control. *Proteobacteria*, *Enterobacteriaceae*, and *Escherichia* were significantly elevated in NASH. The possible mechanism of NASH is the intestinal alcohol-producing bacteria (e.g. *Escherichia coli*) increased, more alcohol could provide a continuous source of ROS to the liver, leading to liver inflammation. Recently, the high-alcohol-producing *Klebsiella Pneumoniae (HiAlc Kpn)* was found in the gut of NASH patients. About 60% of NAFLD patients in a Chinese cohort study are associated with *HiAlc Kpn*. The NAFLD mouse model induced by *HiAlc Kpn* was also successfully established. Through germ-free mice, fecal bacteria transplantation, bacteriophage, and antibiotic treatment experiments, it was proved that *HiAlc Kpn* in intestinal flora was a new cause of NAFLD liver disease, and NAFLD’s etiological theory of “endogenous alcoholic fatty liver disease” was put forward. *HiAlc Kpn* with high ethanol production colonized the intestinal tract and produced a large amount of ethanol, which was transferred into the liver through the portal system, causing dysfunction of the liver (Yuan et al., 2019). It is reported that the mice deficient in junctional adhesion molecule A **(**JAMA) suffered more severe fatty liver and steatohepatitis than control mice when treated mice with a high saturated fat diet. Antibiotics or sevelamer hydrochloride treatment can reduce the severity of fatty liver and steatohepatitis in JAMA deficient mice (Rahman et al., 2016).

### Alcoholic Liver Disease

Alcohol-related liver disease (ALD) accounts for 0.9% of the total deaths worldwide, accounting for 47.9% of the incidence rate of cirrhosis (Pda et al., 2019). Drinking alcohol can increase the imbalance of intestinal bacteria and fungi, leading to the development of disease susceptibility, intestinal barrier function loss, and liver injury (Bajaj, 2019). Cho et al. reported that in ethanol-fed rodents, intestinal cell apoptosis increased with the degradation of tight junction protein (Cho et al., 2018). In addition, the levels of bacterial endotoxins in the blood of alcoholics, alcoholic hepatitis, and liver cirrhosis patients were higher than those of healthy people (Fukui et al., 1991). The gut-derived endotoxins contribute to increased intestinal permeability and alcohol-induced tissue injury (Schnabl and Brenner, 2014). Alcohol could disrupt the tight junctions of epithelial cells, which cause endotoxins translocation and the pathogenesis of ALD. The anti-microbial peptide could be used to treat ALD. When feeding the mice with alcohol, the anti-microbial peptide REG3G expression in the intestinal tissues was reduced (Wang et al., 2016). In addition, when the mice were gavaged with the engineered bacteria which overexpress interleukin 22 (IL22) to induce REG3G expression, the severity of the alcohol-induced liver injury can be reduced (Hendrikx et al., 2019).

The amount of *Bacteroidetes* and *Firmicutes* was reduced, while the *Proteobacteria* was increased in the colon samples of ALD patients (Mutlu et al., 2012). However, a higher abundance of *Streptococci*, *Bifidobacteria*, *Enterobacteria*, and decreased anti-inflammatory *Clostridium leptum* or *Faecalibacterium prausnitziithan* were found in feces of patients with severe alcohol-associated hepatitis than controls (Llopis et al., 2016). The dysbiosis of intestinal flora was observed in alcoholics, *Enterobacteriaceae* significantly increased and *Bacteroidetes* and *Lactobacillus* reduced (Mutlu et al., 2012). Either alcohol withdrawal or probiotic oral supplementation could reduce alcohol-induced dysbiosis (Leclercq et al., 2014). Among alcohol-related liver diseases, alcoholic hepatitis has high mortality almost 20% (Thursz et al., 2015). The gut microbiota composition between patients with severe alcoholic hepatitis (AH) and alcoholic patients without alcoholic hepatitis (non-AH) are significantly different. To study the GM function on the pathology of AH. GM was collected from AH and non-AH groups and transplanted into germ-free mice separately. In the following five weeks, mice were fed an alcohol-containing diet. The non-AH donor GM received group gained more weight than the severe AH donor group. Liver inflammation was more severe in the severe AH donor group (Jefferies et al., 2018). By gut microbiota sequence analysis, in both groups, the abundance of *Bacteroides* was dominant, but in severe AH mice, the number was more marked. *Butyricimonas, Bilophila*, *Clostridium* cluster XIVa and *Alistipes* were also more significantly abundant in the severe AH mice. Large amount of *Parasutterella excrementihominis* was found in non-AH mouse microbiota, but almost absent in severe AH donors, which suggest the bacteria may have a protective effect (Llopis et al., 2016).

### Hepatitis Virus Infection

Hepatitis virus infections include chronic hepatitis B **(**HBV) and chronic hepatitis C (HCV) infection. The gut microbiome is involved in the progression of hepatitis virus-related liver disease. Gut microbiota alterations were significantly associated with liver disease progression. Compared to healthy controls, patients with HBV has a higher abundance of *Veillonella*, *Fusobacteria*, and *Haemophilus*, lower amount *Prevotella* and *Phascolarctobacterium* (Chen et al., 2020) Recent research showed that when comparing the stage 4 HCV patients to healthy controls, the amount of *Bacteroidetes* increased and *Firmicutes* has slightly decreased (at the phylum level), the abundance of *Prevotella*, *Acinetobacter*, *Phascolarctobacterium*, *Veillonella*, and *Faecalibacterium* increased, the genus of *Ruminococcus*, *Clostridium* and *Bifidobacterium* decreased (Aly et al., 2016).

### Primary Sclerosing Cholangitis (PSC)

Primary sclerosing cholangitis is a chronic bile duct disease characterized by inflammation and bile ducts fibrosis, which leads to impaired bile formation or flow. Most patients eventually with a high rate of cirrhosis, portal hypertension, and liver dysfunction (Lazaridis and Larusso, 2016). Currently, there is no effective medical treatment for PSC, and liver transplantation is the only effective option with high cost. Recently, a lot of research has focused on the role of gut microbiota in PSC (Little et al., 2020). Sabino et al. found intestinal microbiota diversity of PSC was decreased, the number of *Enterococcus*, *Fusobacterium*, and *Lactobacillus* significantly increased (Sabino et al., 2016). Another study also found the bacterial diversity was reduced and the mucosa-associated bacteria (an uncultured *Clostridiales* II) was under- represented (Rossen et al., 2015). A further study concluded the number of *Blautia* and *Barnesiellaceae* were increased in the mucosal samples of PSC (Torres et al., 2016). The abundance of *Veillonella* has been proved to be associated with PSC (Iwasawa et al., 2017). *Veilonella* contains genes that can encode amine oxidase, this molecule has important effects on the liver. In various studies, *Veilonella* appears to also have pro-inflammatory features (Van den Bogert et al., 2014). A UK study showed that the increase of *Escherichia*, *Megasphera*, *Lachnospiraceae* and reduction in *Roseburia* and *Prevotella*, *Bacteroides* was almost disappeared in PSC patients (Quraishi et al., 2017). Rühlemann et al. demonstrated eight taxa enriched in PSC compared to healthy controls. An increase of *Proteobacteria* and *Parabacteroides* were newly identified in PSC, and they are bile-tolerant taxons, which are important in cholesterol and bile acid metabolism (Rühlemann et al., 2019). Patients with PSC have a signature characterized by several genera decrease and certain genera increase, these genera could be the biomarkers for PSC and can highly predict PSC (**Table 1**).

### Cirrhosis

Cirrhosis is characterized by hepatocyte loss, fibrous scar thickening, and regenerative nodules (Bhat et al., 2016). NAFLD, ALD, primary sclerosing cholangitis, and hepatitis can progress to cirrhosis (Tsochatzis et al., 2014). Specific bacteria enrichment or decrease were correlated with the severity of cirrhosis (Qin et al., 2014). Spontaneous bacterial peritonitis or worsened liver dysfunction was often found in cirrhosis patients. Quin and colleagues assessed the most extensive metagenome communities of cirrhosis (Qin et al., 2014). They observed severe ecological dysbiosis, and over fifty percent of patients’ abundant taxonomically species were from oral, suggesting that the oral microbiome contributes to cirrhosis processes and severity. Many studies have proved that the fecal microbial community has significant alterations in cirrhosis patients (Bajaj and Khoruts, 2020; Zheng et al., 2020; Trebicka et al., 2021). The abundance of *Fusobacteria* and *Proteobacteria* increased whereas *Bacteroidetes* was significantly reduced in the cirrhosis patients (Chen et al., 2011). Another study showed *Enterobacteriaceae* and *Enterococcus* were significantly increased, the ratio of *Bifidobacterium* and *Enterobacteriaceae* were reduced in cirrhotic patients. The *Bifidobacterium/Enterobacteriaceae* (B/E) reflects the microbial colonization resistance in the intestine.Plasma endotoxin, IL-6, and fecal secretory IgA were negatively correlated with *Bacteroides-Prevotella* group and *Eubacteria* group. The plasma concentration of endotoxin and IL-6 significantly increased in cirrhotic patients (Liu et al., 2012). Bajaj et al. confirmed the association between GM and liver cirrhosis, which showed that GM diversity and symbiosis significantly improved in patients with severe liver cirrhosis after liver transplantation (Bajaj et al., 2017). However, *Bacteroidetes*, *Lachnospiraceae*, and *Ruminococcaceae* were reduced while *Enterobacteriaceae*, *Alcaligenaceae*, *Streptococcaceae*, *Veillonellaceae*, and *Fusobacteriaceae* were increased in cirrhosis patients when comparison with healthy controls (Bajaj et al., 2014b; Trebicka et al., 2021). These findings provide a new approach for the management of liver cirrhosis with an emphasis on gut microbiota regulation.

### Hepatic Encephalopathy (HE)

Hepatic encephalopathy (HE), is a reversible neuropsychiatric impairment caused by impaired liver function. Studies have shown that the decrease of blood hypoxia peristalsis can cause intestinal flora imbalance, thus increasing the release of intestinal ammonia and endotoxin, and inducing hepatic encephalopathy. However, the types of intestinal microbiota involved in this process are not clear. Intestinal dysbiosis, inflammation, and oxidative stress could be observed in the pathogenesis process of HE (Rai et al., 2015). The most possible pathology of HE is the damaged liver cannot convert intestinal nitrogenous toxins into non-toxic substances and excrete them from the body. Other neurotoxins include methionine derivatives such as thiols, phenols and fatty acids, they may act synergistically with ammonia. Food and gut-derived substances such as pseudoneurotransmitters such as octopamine, benzodiazepine ligands and GABA, are elevated in HE and have been blamed for interfering with neurotransmission. However, their significance in HE pathogenesis is unclear. The excess ammonia in the intestine is transferred to the brain and causes brain damage. The urease-producing bacteria which can produce ammonia and endotoxins such as *Proteus* and *Klebsiella* are increased in the intestine (Milosevic et al., 2019), intestinal permeability also are increased and cause bacterial translocation, increase inflammatory response (Bajaj, 2014). *Fecalibacterium*, *Blautia*, *Roseburia*, and *Dorea* were associated with decreased inflammation and good cognition in both HE/non-HE. In contrast, genera over-represented in HE (*Megasphaera*, *Enterococcus*, and *Burkholderia*) were linked to poor cognition and inflammation (Bajaj et al., 2012). When compared to cirrhotic patients without HE, a higher proportion of *Veillonellaceae* was observed in HE, this bacteria was associated with increased inflammatory cytokines (IL-6, TNF-α, IL-2, and IL-13) and poor cognition. The enrichment of the *Alcaligenaceae* family is related to poor cognitive ability. *Proteobacteria* is an opportunistic pathogen that can degrade urea to ammonia, and this gives a reasonable explanation for cognitive loss (Bajaj, 2014). Therefore, it has been assumed that identifying specific fecal microbial characteristics could be used to predict the absence of minimal HE, instead of cognitive testing (Bajaj et al., 2019). These findings suggest that the microbiome composition of HE patients is closely related to cognition and inflammation.

### Hepatocellular Carcinoma (HCC)

Hepatocellular carcinoma (HCC) is the third leading cause of high cancer mortality. Cirrhosis and viral hepatitis may progress to HCC (Cabrera and Nelson, 2010). It is predicted that more than one million individuals will be affected by liver cancer by 2025, HCC accounts for ~90% of liver cancer cases (Llovet et al., 2021). Experiments have provided evidence that gut microbiota plays a role in hepatocarcinogenesis (Yu and Schwabe, 2017). Dipito et al. found that TLR4 deletion can reduce the number and size of HCC, but cannot prevent the incidence of tumors (Dapito et al., 2012). Thus, the TLR4-LPS pathway is not required for HCC initiation but HCC promotion. When the gut barrier was disrupted, LPS from the gut promotes hepatocarcinogenesis through hepatic stellate cells, hepatocyte-tumor compartment, and Kupffer cells (Gupta et al., 2019). Another critical mechanism about the LPS–TLR4 axis promotes HCC formation is through NF­κB­mediated pathway. Activated TLR-4 further triggers inflammatory signaling pathways for NF­κB that induce the production and release of inflammatory cytokines IL-1β and IL-18 (Yu et al., 2010). Moreover, LPS activates TLR4 in HCC cells can enhance their potential invasive ability and induce the transition of epithelial-mesenchymal (Yu and Schwabe, 2017).

Until now, studies conducted on the gut microbiome and different underlying liver cirrhosis, showing that at least some microbial changes have the same features to different aetiologies. Secretion of bile reduced and changes in intestinal secretion of anti-microbial peptides and IgA at end-stage of liver disease. The common alteration of gut microbiota composition in patients with liver cirrhosis includes enrichment of *Veillonella* or *Streptococcus* and decrease of order *Clostridiales* (Bajaj et al., 2014a). In addition, *Escherichia coli* were significantly increased in fecal samples of HCC patients compared to cirrhosis patients without HCC, indicating that hepatocarcinogenesis may attribute to the overgrowth of *E. coli* (Grt et al., 2016). The most common bacteria isolated from tongue swabs of patients with HCC are *Oribacterium* and *Fusobacterium.* On the other hand, decreases of *Lactobacillus*, *Bifidobacterium* and *Enterococcus* were observed in stool samples of HCC patients (Zhang et al., 2012). It was also reported deoxycholic acid (DCA) accelerates the HCC development in obese mice by inducing the senescence-related secretory phenotype (SASP) in HSCs (Yoshimoto et al., 2013). In addition to synergy with DCA, lipoteichoic acid enters the liver through TLR2 and up-regulates the expression of SASP and COX-2 in senescent HSCs (Loo et al., 2017). Both gut microbiome and their metabolites play a vital role in the process of HCC, microbiome-targeted therapeutic modalities for HCC aroused great interest from scholars, and we will discuss possible approaches in detail in the next paragraph.

## Targeting Gut-Microbiota to Manage Chronic Liver Disease

Gut microbiota-related strategies to manage chronic liver disease were summarized in **Table 2**.

**Table 2:** Gut microbiota related strategies to manage chronic liver diseases.

Strategies	Functional substance/Bacterial species	Mechanism of action	Ref.
Diet	Lactulose	acidify the intestinal cavity, inhibit urease producing bacteria, and limit the diffusion of ammonia into blood	(Phongsamran et al., 2010)
	BCAA (leucine, isoleucine and valine)	promote protein synthesis, reduces nitrogen-containing products and prevents the formation of pseudo-neurotransmitters, reduce the progression of liver failure	(Fukushima et al., 2003)
	Thiamine; propose calcium, vitamin D	restore the activity of PDH and KGDH, reducing ROS; reduce cholestasis	(Francis, 2006; Atarashi et al., 2017; Satish and Shaik, 2021)
	SCFAs	activating G-protein coupled receptors or inhibiting histone deacetylase, anti-inflammatory properties of acetate and propionate, and their inhibitory effects on hepatic lipogenesis and lipid accumulation	(Jin et al., 2015; Sahuri-Arisoylu et al., 2016)
	Indole	enhance the tight junction of epithelial cells and mitigate inflammatory responses in the gut	(Beaumont et al., 2018)
	Choline	removing fat from hepatocytes	(Yu et al., 2016)
Antibiotics	*Eubacteriaceae*↑ *Veillonellaceae*↓	reduce the number of toxic metabolites produced by the GM and reduce serum pro-inflammatory cytokine	(Bajaj et al., 2013; Tang, 2014; Ponziani et al., 2016)
Probiotics	*Lactobacillus rhamnosus*	increase in intestinal FFAs concentration	(Shi et al., 2015)
	*Lactobacillus GG*	reduction of alcohol-induced oxidative stress and restoration of barrier function	(Forsyth et al., 2009)
	*Lactobacillus casei Shirota*	may restore phagocytosis of neutrophils by changing IL-10 secretion and TLR4 expression	(Stadlbauer et al., 2008)
	*VSL# 3 (S. thermophilus, B.* *breve, B. bacterium longum, B. infantis, L. acidophilus, L.* *plantarum, L. paracasei and L. delbrueckii subsp. Bulgarius)*	reduced the risk of hospitalization for HE as well as improved Child-Turcotte-Pugh (CTP) and model for end-stage liver disease scores (MELD) in patients with cirrhosis	(Dhiman et al., 2014)
Prebiotics	*pectin*	restore the levels of *Bacteroides*	(Ferrere et al., 2017)
	*Fructooligosaccharide*	promote fatty acid oxidation by up-regulating the expression of peroxidase, inhibit the expression of SREBP-2 in the liver and reduce the accumulation of cholesterol	(Matsumoto et al., 2017)
Synbiotics	*Bifidobacterium longum* and FOS	protect against inflammation and hepatocyte damage	(Malaguarnera et al., 2012)
Fecal microbiota transplant (FMT)	feces bacteria from alcohol resistant mice	FMT prevented alcohol-induced intestinal disorders and fatty hepatitis	(Ferrere et al., 2017)
	fecal bacteria transplantation from health control	improve intestinal flora disorder, enhance intestinal barrier function and reduce liver steatosis	(Zhou et al., 2017)
	fecal bacteria transplantation from health control	effectively reduce alkaline phosphatase in some patients, the ALP level of some patients (30%) decreased, fecal flora diversity of all patients increased	(Allegretti et al., 2019)
	FMT from the donor enriched in *Lachnospiraceae* and *Ruminococcaceae*	serum IL-6 and LPS binding protein were decreased and butyrate/isobutyrate was increased in the FMT group compared with baseline	(Bajaj et al., 2020)

↑ means the abundance of bacteria increase; ↓ means the abundance of bacteria decrease.

### Diet

Many studies focus on dietary modification to optimize the structure of gut microbiota. Higher intakes of branched-chain amino acids (BCAAs, leucine, isoleucine and valine) and vegetable proteins have shown benefits in cirrhosis patients (Fukushima et al., 2003). Serum BCAAs normalization can promote protein synthesis, reduce nitrogen-containing products, and prevent pseudo-neurotransmitters formation, which are important for neurotransmitters’ development. BCAA-enriched diet can reduce the progression of liver failure in patients with advanced cirrhosis, which can reduce the severity and frequency of HE, and improve quality of life.

Vitamin and/or mineral deficiencies can occur in chronic liver disease. Thiamine supplementation should be considered in severely alcoholic patients (Satish and Shaik, 2021). Fat-soluble vitamin supplementation should be considered in conditions of cholestatic (Atarashi et al., 2017). Vitamin K supplementation is only to be considered at high risk of a hemorrhagic situation (Atarashi et al., 2017). Zinc and magnesium supplementation can indirectly improve nutrient intake and nutritional status (Garrett-Laster et al., 1984). Calcium and vitamin D supplementation could be considered in patients with cholestasis and osteoporosis (Francis, 2006). SCFAs regulate liver metabolism and immune function by inhibiting histone deacetylase or activating G-protein coupled receptors, including GPR41, GPR43, GPR109a, and OLFR78. In addition, acetate and propionate have anti-inflammatory properties and inhibit hepatic lipogenesis and lipid accumulation (Sahuri-Arisoylu et al., 2016). Butyrate can modulate the GM composition, increase the expression of glucagon-like peptide-1 receptors (GLP-1R), reduce inflammatory signals and liver oxide damage, ultimately attenuate steatohepatitis (Jin et al., 2015).

Indole can enhance epithelial cells’ tight junction, attenuate indicators of inflammation and intestinal injury. Recently, Beaumont et al. have proved that after feeding the mice with indole, the expression of tight junction protein was up-regulated, and the mice display resistance to liver inflammation (Beaumont et al., 2018). Choline deficiency in diet could lead to reversible hepatic steatosis. Choline has a function in removing fat from hepatocytes (Yu et al., 2016). Metagenomic analysis of the microbial communities in the intestinal tracts of 15 women showed that increased *Gammaproteobacteria* and decreased *Erysipelotrichi* can prevent steatosis with a choline-depleted diet (Spencer et al., 2011).

### Antibiotics

Antibiotics can decrease the total number of gut microbiota, eliminate bacteria with a high ability to translocate and inhibit pro-inflammatory signals arising from the gut ecosystem (Zhang et al., 2015). Rifaximin could be used to treat HE in clinical trials (Kimer et al., 2014). It may increase the number of beneficial bacteria while decreasing the number of harmful bacteria, and also improve beneficial bacteria function. Rifaximin could reduce gut-derived toxins and reduce serum pro-inflammatory cytokines levels (Ponziani et al., 2016). It may also affect gut microbiota metabolites (Bajaj, 2016). The research tested the functions of rifaximin on cirrhotic patients with minimal HE. They analyzed both the structure of gut microbiota and the metabolome of serum and urine. The results showed that after eight weeks of rifaximin treatment, cognition and endotoxemia could be improved. The composition of gut microbiota didn’t change significantly after rifaximin treatment (Kaji et al., 2017), only a slight increase of *Eubacteriaceae* and reduction of *Veillonellaceae* were found in the study (Bajaj et al., 2013). However, the unsaturated and saturated fatty acids in the serum were significantly increased after rifaximin treatment. The results showed rifaximin does not change the overall composition and diversity of gut microbiota, it modifies the metabolites of the bacteria. Rifaximin can also increase the concentration of linoleic and arachidonic acids, which have brain beneficial functions (Tang, 2014).

### Probiotics

Many studies have reported that probiotics have beneficial effects on liver health. Probiotics have functions in improving nutritional stations, repairing mucosal barrier, providing short-chain acids to prevent apoptosis, improving intestinal epithelial viability (Kanauchi et al., 1999). These functions prevent the disturbance of pathogens on tight junctions. *Lactobacillus GG* can improve intestinal oxidative stress, intestinal leakage, and liver injury in the alcoholic steatohepatitis rat model (Forsyth et al., 2009). In patients with alcoholic cirrhosis, probiotics *Lactobacillus casei* Shirota may restore phagocytosis of neutrophils by changing IL-10 secretion and TLR4 expression (Stadlbauer et al., 2008). The effectiveness of probiotics on liver cirrhosis can be divided into improving liver function and preventing pathogens infection, hepatic encephalopathy, and other complications. Intake of VSL#3 daily for six months significantly reduced the risk of hospitalization for HE and improved Child-Turcotte-Pugh (CTP) and model for end-stage liver disease scores **(**MELD) in patients with cirrhosis (Dhiman et al., 2014). The ability of probiotics to modulate GM has therapeutic potential. There is some evidence regarding probiotic usage to treat spontaneous bacterial peritonitis (SBP) and HE. Further research in evaluating gut microbiota and appropriately selected beneficial bacterial strains as treatment modalities should be undertaken (Forsyth et al., 2009).

### Prebiotics

Prebiotics are non-digestible but fermentable food substances fermented by bacteria and help the intestine peristaltic and selectively stimulate the growth of gut bacteria. Some prebiotic foods, like lactulose and pectin, seemed to be promising therapeutic agents to treat liver diseases. Lactulose is an unabsorbable disaccharide, which can acidify the intestinal cavity, inhibit urease-producing bacteria, and limit the diffusion of ammonia into the blood (Phongsamran et al., 2010). The main treatment for HE patients is to target the intestine to reduce colonic bacterial generation and reduce blood ammonia. Pectin can restore the levels of *Bacteroides* and prevent liver damage in rodent models (Ferrere et al., 2017). A meta-analysis of 1309 NAFLD patients showed significant reductions in BMI, liver enzymes, serum cholesterol, and triglycerides after prebiotic treatment. This suggests that prebiotics may improve NAFLD by regulating intestinal flora homeostasis (Loman et al., 2018). Fructooligosaccharide (FOS) can promote fatty acid oxidation by up-regulating peroxidase expression, inhibiting the expression of SREBP-2 in the liver, and reducing the accumulation of cholesterol (Matsumoto et al., 2017). This showed oligosaccharides could reduce bacterial overgrowth and improve alcoholic steatohepatitis by restoring the expression of partial lectin protein reg3g. Lactulose is prebiotic which can promote the growth of *Bifidobacterium* and *Lactobacillus*. Fan et al. used lactulose to treat NASH mice. The results showed that alanine aminotransferase (ALT) and other liver inflammatory indexes in the lactulose treatment group were significantly reduced compared with the non-alcoholic steatohepatitis model group (P < 0.05), which were established by given high-fat diet, but the steatosis of liver cells was not significantly improved, suggesting that lactulose has a certain role in reducing liver inflammation, but it can’t improve hepatocyte steatosis (Fan et al., 2005).

### Symbiotics

Symbiotics consist of a combination of probiotics and prebiotics. Symbiotics have the function of regulating the expression of intestinal flora and related functional genes. It can reduce the colitis reaction and liver inflammatory response, reduce the level of SCFA in feces, enhance tight junction of the intestinal barrier, and improve fatty degeneration of the liver as well as insulin resistance. Meanwhile, symbiotics can reduce the degree of liver fibrosis and LPS in mice. Clinical research indicated that after six months of intervention by *Bifidobacterium longum* and FOS in 66 NASH patients, serum AST, LPS, and inflammatory response transmitters (HOMA-IR), fat denaturation and NASH activity index were significantly reduced (Malaguarnera et al., 2012). Symbiotic treatment could be an ideal approach to treat liver diseases since they can protect against inflammation and hepatocyte damage through probiotics and prebiotics. The mechanism of symbiotic protection has not yet been fully clarified. Potential mechanisms of symbiotic effect on liver disease include gut microbiota structure and microbiota metabolite alteration, which have anti-inflammatory and immunomodulatory effects. There are no adverse effects that have been reported with this approach.

### Fecal Microbiota Transplant (FMT)

Fecal microbiota transplantation (FMT) means transplanting bacteria from the feces of healthy individuals into the recipient through a series of routes (Xu et al., 2021). FMT can restore the health of intestinal flora, further reduce the transport of endogenous ethanol, endotoxin, and other metabolites to the liver, reduce the damage of metabolic substances to the liver, and also effectively reduce the inflammatory response and the expansion of liver cells. Compared with the oral probiotics method, FMT can significantly improve intestinal bacteria disorder, and FMT is the most effective method to restore intestinal microecological balance currently.

FMT has been studied more deeply in recent years. The possible mechanism of FMT involves establishing beneficial microbes in the gut and the production of anti-microbial substances. In Ferrer’s study, they transplanted fecal bacteria from alcohol resistant mice to alcohol sensitive receptor mice and found that FMT prevented alcohol-induced intestinal disorders and fatty hepatitis in the liver to some extent. The number of *Bacteroides* in alcohol-sensitive mice decreased, and the number of *Actinobacteria* and *Firmicutes* increased. Alcohol-sensitive mice had fifty percent fewer *Bacteroides* than alcohol-resistant mice (Ferrere et al., 2017). Zhou et al. used fecal bacteria transplantation to treat NAFLD mice caused by a high-fat diet. The results showed that fecal bacteria transplantation could improve intestinal flora disorder, enhance intestinal barrier function and reduce liver steatosis (Zhou et al., 2017). At present, FMT has successfully treated *Clostridium difficile* infection patients in clinical and promoted the recovery of intestinal ecological balance. Simultaneously, the clinical results showed that the improvement effect was better than that of standard antibiotics. A clinical trial showed that FMT showed good safety in 10 patients with primary sclerosing cholangitis complicated with IBD, and it could effectively reduce alkaline phosphatase in some patients. Liver enzymes, fecal microflora, and fecal metabonomics were analyzed at 1, 4, 8, 12, and 24 weeks after FMT. The mean baseline level of ALP was 489U/L. FMT rarely has adverse events. The ALP level of 3 patients (30%) decreased by more than 50%; The fecal flora diversity of all patients increased after transplantation, and the successful colonization of donor flora in patients was related to the decrease of ALP (Allegretti et al., 2019).

Therefore, FMT can be used as an effective method to treat and prevent chronic liver disease. The preliminary basic research shows that FMT can restore the probiotics in the intestinal ecosystem (Craven et al., 2020; Lechner et al., 2020; Liu et al., 2021). Recently one randomized, double-blind trial was performed, patients with ALD‐related cirrhosis were randomized to receive FMT from a donor enriched in *Lachnospiraceae* and *Ruminococcaceae* or one placebo. On day 15, craving, urinary ethyl glucuronide/creatinine, cognitive and psychosocial quality of life was largely reduced in 90% of the FMT group and 30% of the placebo group (P = 0.02), while serum IL-6 and LPS-binding protein were decreased and butyrate/isobutyrate was increased in FMT group compared with baseline, but not in the placebo group (Bajaj et al., 2020). Therefore, fecal bacteria transplantation could be a valuable and safe treatment for cirrhosis. The advantage of FMT is that it can rebulid the whole dysbiotic intestinal environment, but the best optimal route of administration, the duration of treatment and the durability of reaction still need to be determined. There is an urgent need for large-scale and high-quality research in this field in order to evaluate the most effective method to achieve rebiosis.

## Conclusions

The gut microbiota plays an important role in the pathogenesis of chronic liver diseases, including NAFLD, ALD, Hepatitis Virus Infection, PCS, cirrhosis, and HE. The mechanism of connection between liver diseases and the intestine is poorly understood. Intestinal permeability is closely related to chronic liver disease. The increase of intestinal permeability will lead to the release of intestinal inflammation factors and cause flora dysbiosis, which contributes to liver disease development (Fukui, 2019). The liver can communicate with the gut through bile acids circulation. The binding of BAs to FXR can induce anti-microbial peptides to produce, which can inhibit gut microbial overgrowth and improve gut barrier function. Choline assists the liver in excreting VLDL particles, preventing the accumulation of triglycerides in hepatic steatosis. Intestinal bacteria can convert choline into TMA. TMA can be transferred to the liver and converted to TMAO, which is associated with liver injury. Gut microbiota also can produce SCFAs including butyrate, propionate, acetate, etc. Butyrate can reduce intestinal permeability and subsequent liver injury induced by alcohol. Since the gut microbiota places an important role in the progress of the chronic liver disease, we believe the gut-microbiota-targeted interventions could be used to regulate the intestinal flora community and manage chronic liver diseases.

However, a comprehensive understanding of the interactions between microbes and liver diseases is still not clear. Although animal models help to elucidate many important mechanistic pathways in the etiology of liver diseases. From animal models to human models requires well-designed, large-scale clinical trials spanning multiple disease aetiologies and patient characteristics. With the increasing recognition of the role of the microbiome in the development, prognosis, and treatment of liver diseases, we emphasize the need for a focus on the microbiome to effectively address the socioeconomic burden of this type of liver disease. Furthermore, it is critical to use animal models that mimic human disease as closely as possible.

## Author Contributions

JL contributed to manuscript writing. DY, XW, QZ, and LN critically reviewed the manuscript. PA critically edited the English in the manuscript. LS supervised the whole process and reviewed the manuscript. All authors contributed to the article and approved the submitted version.

## Funding

This research was financially supported by the Natural Science Foundation of Shanghai (20ZR1424600), the National Natural Science Foundation of China (81773616), Shanghai Excellent Technology Leader Program (17XD1423200), Shanghai Municipal Public Health System Construction Three-Year Action Plan (2020-2022) (GWV-10.1-XK01), National Natural Science Foundation of China (82100666), National Natural Science Foundation for Young Scientists of Jiangsu Province (BK20200906), and Shanghai Education Development Project for Industry-University-Research Practice (A3-0200-21-311007-34).

## Conflict of Interest

The authors declare that the research was conducted in the absence of any commercial or financial relationships that could be construed as a potential conflict of interest.

## Publisher’s Note

All claims expressed in this article are solely those of the authors and do not necessarily represent those of their affiliated organizations, or those of the publisher, the editors and the reviewers. Any product that may be evaluated in this article, or claim that may be made by its manufacturer, is not guaranteed or endorsed by the publisher.
